# Genetic investigations in cerebral palsy

**DOI:** 10.1111/dmcn.16080

**Published:** 2024-08-29

**Authors:** Anna P. Basu, Karen Low, Thiloka Ratnaike, David Rowitch

**Affiliations:** ^1^ Population Health Sciences Institute, Newcastle University Newcastle upon Tyne UK; ^2^ Paediatric Neurology Great North Children's Hospital Newcastle upon Tyne UK; ^3^ Centre for Academic Child Health University of Bristol Bristol UK; ^4^ Department of Clinical Genetics University Hospitals Bristol and Weston NHS Trust Bristol UK; ^5^ Department of Paediatrics University of Cambridge Cambridge UK; ^6^ Paediatrics Colchester Hospital, East Suffolk and North Essex NHS Foundation Trust Colchester UK

## Abstract

The original description of cerebral palsy (CP) contained case histories suggesting that perinatal environmental stressors resulted in brain injury and neurodevelopmental disability. While there are clear associations between environmental impact on brain development and CP, recent studies indicate an 11% to 40% incidence of monogenic conditions in patients given a diagnosis of CP. A genetic diagnosis supports the delivery of personalized medicine. In this review, we describe how the Wnt pathway exemplifies our understanding of pathophysiology related to a gene variant (*CTNNB1*) found in some children diagnosed with CP. We cover studies undertaken to establish the baseline prevalence of monogenic conditions in populations attending CP clinics. We list factors indicating increased likelihood of a genomic diagnosis; and we highlight the need for a comprehensive, accurate, genotype–phenotype reference data set to aid variant interpretation in CP cohorts. We also consider the wider societal implications of genomic management of CP including significance of the diagnostic label, benefits and pitfalls of a genetic diagnosis, logistics, and cost.

## Graphical abstract

Evidence emerging from recent studies indicates an 11% to 40% incidence of monogenic conditions that may have been misdiagnosed as cerebral palsy (CP). Here, we consider the potential impact of genomic diagnosis in patients with a clinical diagnosis of CP.

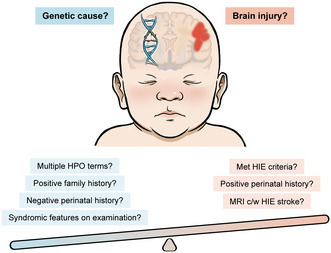

Plain language summary: https://onlinelibrary.wiley.com/doi/10.1111/dmcn.16151

**Abbreviations**
HIE: hypoxic–ischaemic encephalopathy
HPO: Human Phenotype Ontology
MIM: Mendelian Inheritance in Man
WGS: whole‐genome sequencing
Wnt: wingless related integration site

**What this paper adds**

- The *Wnt* pathway provides an example of our understanding of pathophysiology related to a specific genetic disorder seen in some children diagnosed with CP (*CTNNB1*).
- There are potential benefits, pitfalls, and logistic considerations to negotiate before instigating widespread genomic testing in those with CP.
- We recommend development of a comprehensive and accurate genotype–phenotype CP reference dataset.

Genomic testing provides the opportunity to improve diagnosis and practise personalized medicine, tailoring explanations, monitoring, and interventions to the individual on the basis of knowledge of the implications of a specific genetic finding. It is used in paediatric neurology in contexts such as early‐onset epilepsy to look for an underlying monogenic cause, or repurposing anticonvulsant therapy. However, such testing has not been routinely adopted for use in children with cerebral palsy (CP), despite increasing evidence of a genetic contribution to this condition in many cases. In this review, we discuss the potential role of genomic testing in children with a working diagnosis of CP. We cover the impact of genomic tests on the diagnostic odyssey and personalized treatments. We review (1) CP viewed in the context of classically associated neonatal brain injuries, followed by an overview of the *Wnt* (wingless related integration site) pathway (implicated in neonatal white matter injuries) as one specific example of the role of genetic factors; (2) a summary of recent pivotal papers providing insight into the genetic aetiology of conditions with a working diagnosis of CP, as well as perspectives relevant to clinical care pathways; and (3) challenges and benefits of genomic diagnosis in the context of clinical care for those with CP.

The initial description of neurodevelopmental symptoms that later become known as CP is ascribed to William John Little in 1861. His seminal paper, ‘On the influence of abnormal parturition, difficult labour, premature birth, and asphyxia neonatorum on the mental and physical condition of the child, especially in relation to deformities’, published in the *Transactions of the Obstetrical Society of London*, led to recognition that adverse conditions in the perinatal and neonatal period can result in neurodevelopmental delay and motor impairment.^1^

CP, as defined by Rosenbaum et al., describes ‘a group of permanent disorders of the development of movement and posture, causing activity limitation … attributed to non‐progressive disturbances … in the developing fetal or infant brain. The motor disorders of CP are often accompanied by disturbances of sensation, perception, cognition, communication, and behaviour; by epilepsy, and by secondary musculoskeletal disorders’.^2^ The term is descriptive not aetiological and stands even if an underlying aetiology (genetic or otherwise) is subsequently found.^3^ This definition is subject to current scrutiny and potential revision, with inclusion of the views of those affected.^4^

It is well recognized that maldevelopment or injury/insult to the developing brain in utero or as a neonate or infant (e.g. perinatal complications of placental abruption and disruption of neonatal circulation leading to hypoxic–ischaemic encephalopathy [HIE]) can lead to CP. In the 1960s the specialties of obstetrics and neonatology helped decrease rates of CP through advances in care including more effective infant resuscitation. Associated with new expertise came the practice of resuscitating infants born preterm at younger ages (22 weeks' gestation in many centres). While rates of CP in high‐income European countries and Australia have fallen,^5^, ^6^ the birth prevalence remains high in low‐ and middle‐income countries. Birth prevalence of CP in high‐income countries is estimated as 1.6 per 1000 live births but is over twofold greater in some low‐ and middle‐income countries.^7^

Antenatally, chromosomal disorders, congenital infections such as cytomegalovirus, and fetal coagulation disorders are examples of conditions leading to CP. Common types of premature neonatal brain injury include intraventricular haemorrhage, gliosis, and periventricular leukomalacia (Figure 1a, adapted from Silbereis et al.^8^); known sequelae respectively include ventriculomegaly and hypomyelination.^9^ HIE at term damages the cortex, deep grey matter, and white matter tracts.^10^ Another condition resulting in CP is neonatal stroke, with an estimated birth prevalence of 1:1100 in term‐born infants^11^ (Figure 1b, adapted from Silbereis et al.^8^). Injuries/insults to the developing brain beyond the neonatal period can also lead to a diagnosis of CP.^12^

**FIGURE 1:**
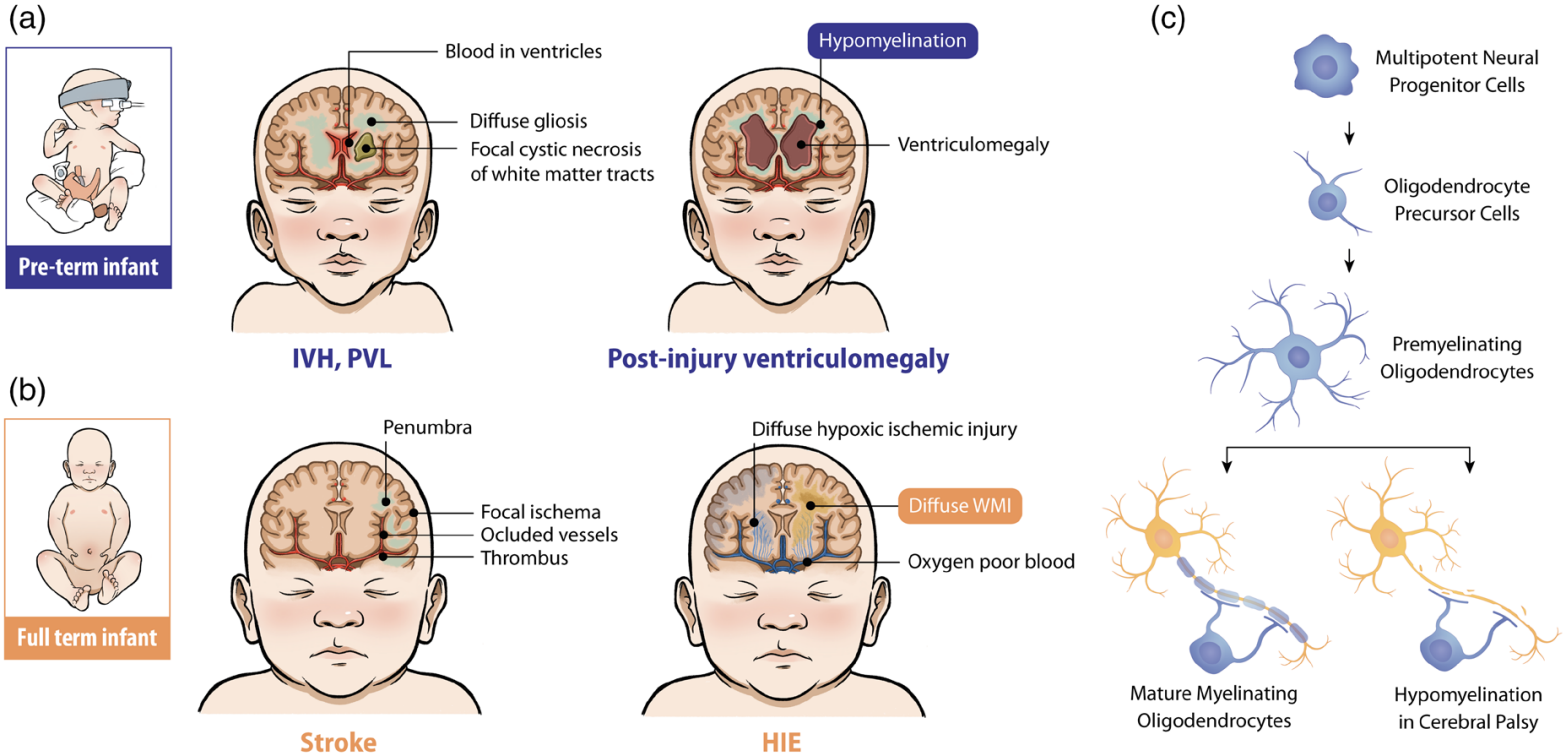
Common types of neonatal brain injury and impact on white matter associated with cerebral palsy. (a,b) Illustration of brain injuries commonly affecting extra‐low‐birthweight infants born (a) preterm and (b) at term (both parts adapted from Silbereis et al.^8^). (a) IVH results from germinal matrix bleeding into the ventricles, sometimes extending into the brain parenchyma. Additionally, there is a high incidence of periventricular leukomalacia (a type of white matter injury) comprising cystic necrosis of white matter tracts and/or diffuse gliosis. Long‐term sequelae of brain injury in extra‐low‐birthweight infants are shown, including hypomyelination resulting from failure of lesion repair and ex vacuo ventriculomegaly resulting from significant loss of brain parenchyma. (b) Common brain injuries in infants born at full term. Neonatal stroke in which a region in one hemisphere of cortex is typically affected. HIE is global hypoxic–ischaemic injury to the brain, including neurons of the cortical plate and basal ganglia as well as white matter tracts. (c) Scheme to show sequence of oligodendrocyte development from multipotent cell to precursor pre‐myelinating oligodendrocytes that normally supply myelin segments to maturing axons. In neonatal white matter lesions, oligodendrocyte precursors blocked in differentiation that fail to myelinate axons are observed, a component leading to hypomyelination in cerebral palsy. Abbreviations: HIE, hypoxic–ischaemic encephalopathy; IVH, Intraventricular haemorrhage; PVL, periventricular leukomalacia.

Advances in neurosciences and genetics promise to enhance our understanding of basic pathophysiology of CP. For white matter injury, developmental biology of the oligodendrocyte lineage has provided mechanistic insights into the nature of injuries resulting in hypomyelination. The discovery of *Olig* genes (*Olig1*, *Olig2*) provided biomarkers to assess the earliest stages of lineage development and differentiation block imposed by injury.^13^ Work from Kinney's laboratory showed that differentiation block at the pre‐myelinating to myelinating stage of oligodendrocyte lineage development was characteristic of neonatal white matter injury^14^ (Figure 1c).

Rowitch's laboratory identified the *Wnt* pathway as an inhibitor of the transition from pre‐myelinating to myelinating oligodendrocyte lineage and human pathological studies demonstrated *Wnt* pathway activation in neonatal HIE and preterm white matter injuries.^15^, ^16^, ^17^ The *Wnt* pathway serves as an exemplar for inhibitors to oligodendrocyte lineage differentiation which may help explain hypomyelination in CP. We highlight it here because mutations of the *Wnt* pathway component *β*‐catenin (*CTNNB1* gene) are among the most common found in patients with CP.^18^ We acknowledge that there are other genetic contributors to outcome after neonatal brain injury.

Originally discovered as both a segment polarity gene in *Drosophila* and in mammalian proto‐oncogene, the wingless/*Wnt* pathway is fundamental to development of all organisms, with important roles in oncogenesis, particularly colon cancers.^19^ In the absence of *Wnt* ligand, the critical downstream effector β‐catenin is degraded by a complex including Axin, GSK3β, and adenomatous polyposis coli proteins (Figure 2). In the presence of ligand, the inactivation complex is disrupted, allowing accumulation of β‐catenin, which can then translocate to the nucleus associated with TCF7L2/LEF1 transcription factors resulting in activation of target genes such as *Axin2*.^16^ In colon cancer, the *Wnt* pathway threshold levels are extremely high, owing to an inability to inactivate and degrade β‐catenin, which causes unrestricted signalling. Fancy et al. showed high threshold signalling in human neonatal white matter lesions, which, on the basis of mouse modelling, is expected to delay/inhibit oligodendrocyte lineage differentiation.^19^ Variants of β‐catenin, encoded by the gene *CTNNB1*, are common in several reports of children clinically diagnosed with CP (Figure 3):^20^ these are predicted to either activate or inhibit the *Wnt*pathway, which in turn is likely to impact development of neurons, myelinating oligodendrocytes, and/or brain vasculature.

**FIGURE 2:**
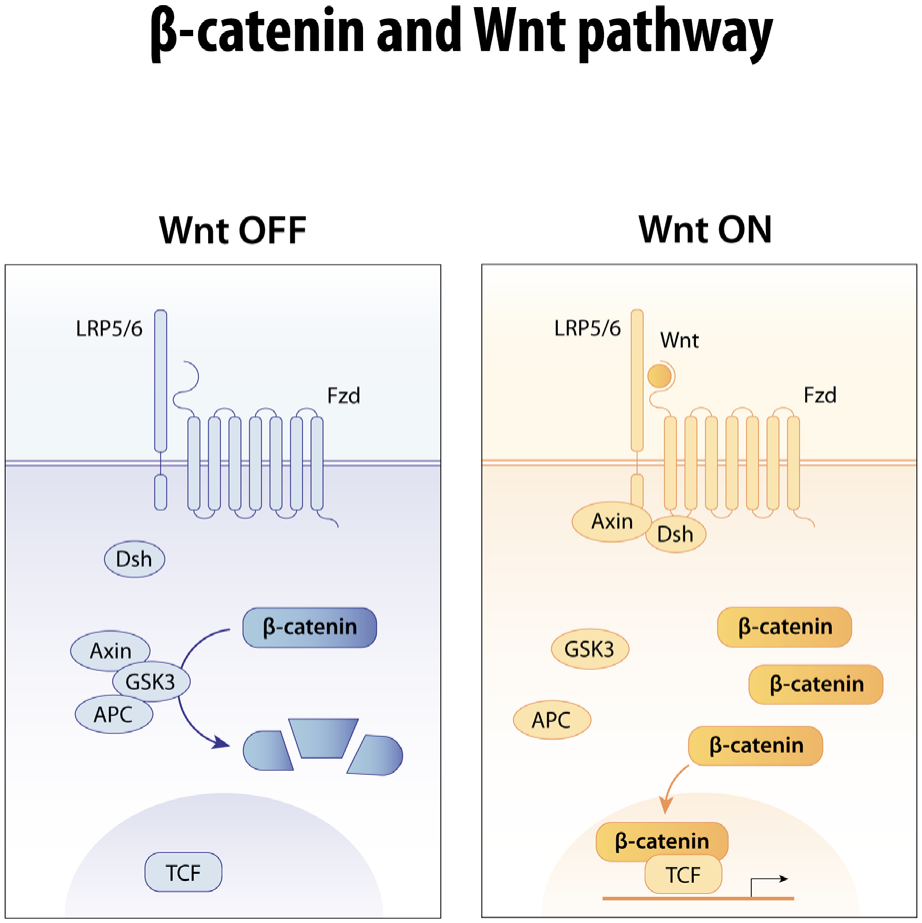
*Wnt* pathway. This pathway is highly conserved during evolution. In the ‘off’ state (left) in the absence of *Wnt* ligand, β‐catenin undergoes degradation through a complex of Axin, GSK3β, and adenomatous polyposis coli proteins. The presence of *Wnt* ligand (‘on’ state, right) activates a coreceptor complex comprising LRP5/6 and Frizzeled to stabilize β‐catenin in the cytoplasm, which translocates to the nucleus to associate with T‐cell/lymphoid enhancer transcription factors to activate target genes. Analysis of pathological lesions in cerebral palsy reveals oligodendrocytes with *Wnt* pathway activation that impedes their differentiation. Variants of β‐catenin are frequently observed in people with cerebral palsy phenotypes: these are predicted to either activate or inhibit the *Wnt* pathway, which is critically important in the development both of neurons and of myelinating oligodendrocytes.

**FIGURE 3:**
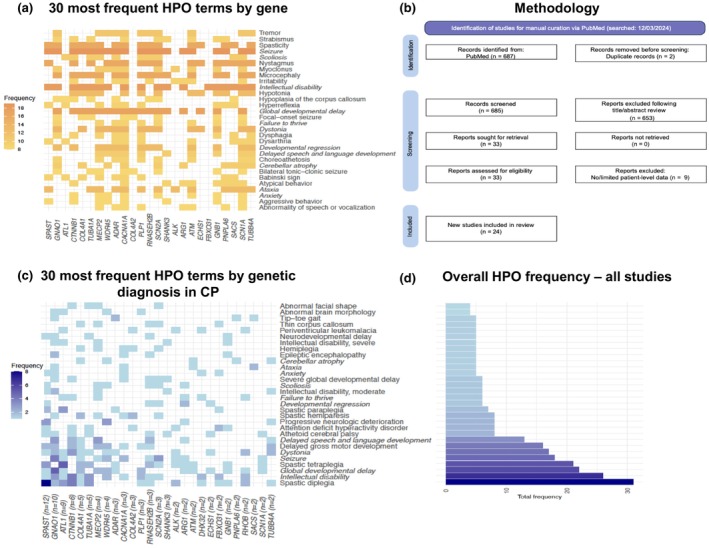
HPO terms associated with genetic diagnoses in CP. (a) The 30 most frequently found HPO terms for 25 genes associated with cohorts of patients with CP (identified as in [b]; downloaded from https://hpo.jax.org/ on 18th February 2024). Matching HPO terms with (c) are shown on the *y*‐axis in italic type. (b) PRISMA flowchart for the PubMed literature review undertaken to identify 24 peer‐reviewed articles, in the past 5 years, with patient‐level phenotype data for CP case series and cohort studies. (c) The 30 most frequent HPO terms documented in patient‐level data across the 24 studies identified in (b), across 27 genes. The number of patients per gene is shown in parentheses next to gene name on the *x*‐axis. The matching 11 HPO terms with (a) are shown in italic type on the *y*‐axis. (d) Overall frequency of the top 30 HPO terms shown in (c) (excluding clinical modifier terms such as onset) across all publications, with ‘spastic diplegia’ being most frequent, and ‘abnormal facial shape’ being least frequent. Abbreviations: CP, cerebral palsy; HPO, Human Phenotype Ontology; PRISMA, Preferred Reporting Items for Systematic Reviews and Meta‐Analyses.

## RECENT GENETIC INSIGHTS INTO CP

Investigations into CP initially used genome‐wide association studies and later exome sequencing to investigate potential genetic contributors. These could comprise monogenic disease mimicking CP but with distinct clinical features (e.g. leukoencephalopathies, and neurodegenerative, neurometabolic, and neuromuscular disorders); monogenic disorders with clinical presentations compatible with CP; or genetic vulnerabilities leading to more significant brain damage in the context of injury.^21^ Exome sequencing captures the genetic sequences of coding regions of the human genome, and studies from several countries have shown that a proportion (11% to more than 40%) of people with a diagnosis of CP have a monogenic pathological DNA variant.^22^, ^23^, ^24^, ^25^

Whole‐genome sequencing (WGS) forms the mainstay of genomic testing in high‐income settings. All paediatric WGS combines a panel test and a range of gene‐agnostic approaches including de novo variant analysis (where trio samples are available), Exomiser top‐ranked hits, and copy‐number analysis.^26^ Clinicians determine eligibility for genomic testing on the basis of the patient's phenotype. For example, in the UK each test indication is given a particular ‘R‐code’ in the national genomic test directory (https://www.england.nhs.uk/genomics/the‐national‐genomic‐test‐directory).^27^ Each R‐code is associated with a specific gene panel consisting of genes selected as highly associated with that phenotype by a group of experts (deemed ‘green’ genes). Lists of genes for each test indication are available online (https://panelapp.genomicsengland.co.uk/).^28^ In the current iteration of the Genomic Test Directory there is no specified R‐code for CP. The Directory is reviewed periodically: clinicians/scientists can submit applications for reviews of eligibility criteria and propose new indications. The scientific evidence and clinical/service implications for these applications are centrally reviewed. Many genomic laboratory hubs are currently experiencing significant delays in test turnaround times.

A systematic review and meta‐analysis of exome sequencing among 15 study cohorts found a diagnostic yield of 35% (95% confidence interval 27–45%) in cryptogenic CP compared with 7% (95% confidence interval 4–12%) in cohorts with known risk factors for CP.^23^ Variants in 23 genes were identified across the cohorts, with *CTNNB1* (Wnt pathway) being the most frequently identified. Most of these genes are associated with additional phenotypes that should serve as ‘red flags’ for a genetic diagnosis. Distinguishing features in *CTNNB1*‐related syndrome include exudative retinopathy, significant postnatal microcephaly, growth restriction, and congenital heart disease (Mendelian Inheritance in Man [MIM] #615075).^18^, ^23^, ^29^, ^30^ The meta‐analysis revealed other conditions with distinguishing features, for example progressive lower limb weakness and spasticity (*SPAST*, MIM #182601);^20^ or a severe Rett‐like phenotype with most individuals being non‐verbal and non‐ambulant (*FOXG1*, MIM #613454). Other examples include cortical visual impairment, seizures, peripheral neuropathy, ataxia, and dystonia associated with *KIF1A* (NESCAV syndrome, MIM #614255); and X‐linked specific dysmorphic features with marked speech delay in *DDX3X* (MIM #300598). Features of *DYRK1A* (MIM #61404) include dysmorphism, intrauterine growth restriction, microcephaly, and urogenital and ophthalmic abnormalities. *TCF4* (also known as *TCF7L2*, MIM #610954) causes Pitt–Hopkins syndrome associated with recognizable dysmorphic features, significant intellectual disability, gastrointestinal abnormalities, and episodic hyperventilation.^27^, ^29^

How do genomic findings associate with phenotype terms in CP? We conducted a PubMed systematic search (detailed in Appendix S1) of patient‐level phenotype data in the form of Human Phenotype Ontology (HPO) terms for 199 people with CP who were given a monogenic disease diagnosis. The HPO is the most comprehensive resource available for computational deep phenotyping and is the de facto standard in the field of rare disease, for clinical descriptions, computable disease definitions, and to aid genomic diagnostics. We compared this data set with available reference HPO annotations per gene (accessed 18th February 2024)^31^ (Figure 3a,b and Appendix S1). HPO terms such as spastic diplegia, spastic tetraplegia, and spastic hemiparesis occurred frequently. Terms such as athetoid cerebral palsy, dystonia, progressive neurological deterioration, global developmental delay, and intellectual disability were also significantly enriched in the patient‐level data set (Figure 3c,d). Enriched terms were defined as those that were more prevalent in the published data set than would be expected on the basis of general associations in the HPO database. It is not surprising that people with a monogenic disease diagnosis and CP have a higher frequency of HPO terms related to clinical features of CP than an unselected group of people with the same monogenic disease diagnosis. The term ‘progressive neurological deterioration’ was enriched in the patient‐level data set. CP is a non‐progressive condition, so this finding indicates that patients misdiagnosed as having CP were included in the published data. This highlights the need for a comprehensive and accurate genotype–phenotype reference data set to aid variant interpretation in CP cohorts.^32^

Magnetic resonance imaging (MRI) brain findings can provide insight into the likelihood of a genetic cause of a CP phenotype (Figure 3).^33^ For example, disorders of hypomyelination and certain neuronal migration defects are highly indicative of genetic causes although some findings such as polymicrogyria can be genetic or acquired. A normal MRI brain is also a ‘red flag’ for an underlying genetic/metabolic cause such as hereditary spastic paraparesis or dopa responsive dystonia, for example in a child with a bilateral presentation and typically no history of preterm birth.

Findings in published reports (detailed in Appendix S1 and summarized in Table 1) suggest certain ‘red flags’ for WGS testing. As an example of such a study, Janzing et al.^34^ undertook a systematic search to identify genes associated with CP and compare the clinical characteristics of this group with those of large groups of patients with CP. Dyskinesia and the absence of spasticity were strongly associated with genetic causes. Intellectual disability, the absence of a history of preterm birth, and bilateral symptoms were moderately associated with genetic causes (the last of these probably because of the high incidence of perinatal stroke as an acquired cause of unilateral CP).^34^

**TABLE 1:** A framework for concerning clinical presentations, additional history, and investigation findings that could trigger further clinical evaluation and genetic testing.

Clinical presentation	Further history	Additional investigations
Multiple features co‐occurring: Developmental regression. Progressive neurological symptoms/deterioration. Movement disorder, for example ataxia, dyskinesia, dystonia, athetosis. Global developmental delay or intellectual disability. Epilepsy. Additional systems involved, for example congenital heart disease and retinopathy (*CTNNB1*). Severe psychiatric/behavioural concerns.	Perinatal: Intrauterine growth retardation (*DYRK1A*). Multiple miscarriages. Anomaly scan abnormalities. Postnatal failure to thrive including feeding difficulties (*TCF4*, *MECP2*).	MRI brain imaging: Corpus callosum abnormalities (*TUBA1A*). Cerebellar atrophy (*CACNA1A*). Basal ganglia abnormalities not consistent with HIE (*TUBA1A*, *ADAR*). Progressive changes suggesting neurodegenerative conditions (*ADAR*). Abnormal myelination (*ADAR*). Normal MRI brain (e.g. hereditary spastic paraplegia). Targeted investigations showing abnormalities CSF, metabolic blood and urine investigations.
Physical examination: Syndromic features (*DDX3X*, *DYRK1A*). Height ± 2 SD (out of keeping with family). Microcephaly not explained by HIE (*DYRK1A*). Abnormal gait and movement pattern indicating a cerebellar pathology or a progressive movement disorder (*MECP2*). Scoliosis (*CTNNB1*). Hypotonia (*COL4A1*). Progressive spasticity and weakness in lower limbs (*SPAST*).	Contributing family history: Other children with similar phenotypes. Other affected family members with other phenotypes (may be part of a spectrum). Consanguinity.	

Examples of genes associated with the various features are given in brackets. It is essential to note that many of those listed (shaded grey in the table) are seen commonly in cerebral palsy and other paediatric cohorts and so, by themselves, are not differentiating factors. It is likely that, for all these features, more severe presentations are more significant in the assessment.

Abbreviations: CSF, cerebrospinal fluid; HIE, hypoxic–ischaemic encephalopathy; MRI, magnetic resonance imaging; SD, standard deviation.

In particular, co‐occurrence of multiple severe phenotypes may warrant further investigation, as individually many features are non‐specific. It is crucial to concentrate on accurate phenotyping, careful clinical assessment, and to re‐evaluate when new phenotypic information becomes available. Phenotype‐driven genetic risk calculators may be an area of future research.

## SOME OUTSTANDING QUESTIONS ON GENOMIC TESTING IN CP

While it has been proposed that all people with CP should be offered genetic testing, this does not constitute standard care in many countries. Important questions remain for consideration before any decision about changes in practice.

### Is it CP?

A diagnostic label can have significant roles in social, administrative, scientific, and clinical contexts.^35^ A diagnostic label can (rightly or wrongly) affect access to support and services (administrative context): it is critical that access should not worsen following any subsequent aetiological clarification. A change in diagnostic label after living with CP as part of one's identity could be confusing, disorienting, and unnecessary (social context). There is a responsibility to use the CP label correctly in the first place. Thorough history‐taking (including family history), examination, and appropriate investigations help avoid misdiagnosis in conditions that might be considered as CP mimics, for example neuromuscular disorders, neurodegenerative/neurometabolic conditions, or leukoencephalopathies (clinical context).^21^ Taking a detailed family history is crucial: for example *COL4A1/2* mutations^36^ show worsening CP severity in successive generations.

For those with CP who subsequently acquire a genetic diagnosis, it is helpful to draw parallels with ‘the epilepsies’, acknowledging the range of possible causes of CP and levels or axes of description of these in any one person.^37^ The use of ‘common data elements’ as developed by the International CP Genetics Consortium would help improve case description. Furthermore, we recommend that studies investigating genetic factors associated with CP adopt a rigorous and standardized approach to case ascertainment. Guidelines from the Surveillance of Cerebral Palsy in Europe group include waiting until the child is at least 4 years of age before officially including as CP in registry data, to reduce the likelihood of inadvertently including progressive disorders. The Surveillance of Cerebral Palsy in Europe diagnostic decision tree also excludes children with generalized hypotonia and acknowledges the challenges of distinguishing between ataxic CP and progressive cerebellar disorders.^38^ Unfortunately, compliance with these guidelines in some genetic studies is limited, with implications for the conclusions that can be drawn (scientific context).^39^

In summary, correct clinical assessment and classification as CP, then monitoring for signs of progression, are crucial. If a genetic aetiology is subsequently found, the CP label should remain if compatible with the clinical presentation.^38^

### What constitutes a ‘CP gene’?

Genes associated with CP risk (including those coding for several interleukins and tumour necrosis factor) are more challenging in terms of explanation, genetic counselling, and implications for management than monogenic disorders. Identifying monogenic disorders can also be more clearly justified in terms of understanding prognosis, recurrence risk in future pregnancies, and in some cases targeting specific interventions or treatments, for example with metabolic disorders (e.g. *ARG1*).^40^ The emerging literature on neonatal encephalopathy with suspected hypoxic–ischaemic encephalopathy is illustrative, where genes associated with neonatal encephalopathy are being identified.^41^ If we investigate for genes associated with CP risk/neonatal encephalopathy with suspected hypoxic–ischaemic encephalopathy, we broaden our criteria for testing beyond those with a clear family history, dysmorphic features, etc., to include all those with suspected neonatal HIE. This would have cost implications and, in the short term, could raise more questions for families than it might answer.

### Can a genetic diagnosis be disadvantageous?

Finding a genetic cause or contributor to CP has potential medicolegal implications and could hypothetically undermine litigation against the medical provider for damages. This calculation might influence a family's decision to pursue genetic diagnosis. Such considerations must be balanced against the potential benefits. For example, understanding a de novo genetic cause might provide relief from guilt experienced by parents who felt they ‘did something wrong’ that led to their child having CP. Genetic findings can also lead to individualized care, which is expected to promote better outcomes: this is a central tenet of the concept of personalized medicine.^42^, ^43^, ^44^ Examples include referral for genetic counselling, increased accuracy of prognostic information, and screening for specific associated disorders such as exudative vitreoretinopathy in CTNNB1 for which laser therapy may be required. Enriching phenotypes in monogenic disorders to improve clinical care is currently the focus of the GenROC study—a cohort study of 500 children with neurodevelopmental genomic disorders.^45^

There are potential disadvantages if genetic testing is not pursued, including missed opportunities to diagnose a progressive condition early, intervene in a treatable condition, participate in a clinical gene therapy or precision drug repurposing trial, or plan future pregnancies. These considerations highlight the importance of providing high‐quality information about the potential implications of genetic testing, with time to ask questions and make an informed decision about whether to proceed. We should also continue to seek the opinions of those with experience of CP about diagnostic processes and their implications.^46^

### Are there benefits from a genomic diagnosis?

While several ongoing studies are exploring the practical benefits and acceptability of genetic diagnosis in people with CP, more research is needed. This is especially relevant given the wide range (11–40%) of diagnostic returns reported to date,^22^, ^23^, ^24^, ^25^ reflecting in part genetic differences at the population level. The examples below were presented at the 2024 European Society for Human Genetics conference.

The 100,000 Genomes Project recruited around 1500 people with a diagnosis of CP: a monogenic diagnosis was identified in more than 32% (personal communication, HH and TL, University College London), in keeping with published studies. The Cambridge Next Generation Children's Project included 15 patients with CP, four (27%) of whom had a monogenic condition.^47^ The ongoing ‘NeuralNET for Cerebral Palsy study’ is using whole‐genome trio diagnosis in 100 patients with CP, recruited from three sites. A participatory exercise with survey respondents indicated 16 out of 18 (~90%) found genomics acceptable if it helped to understand the cause of the condition or improved treatment. The NeuralNET study is expected to complete in 2024 and will provide information on diagnostic returns. It will also explore clinicians', patients', and families' views on the acceptability of genetic testing for CP.

### Which patients with CP should have genetic testing and who should decide?

Chopra et al.^22^ undertook whole‐exome sequencing on a cohort of 50 probands. They split their cohort into cryptogenic CP, non‐cryptogenic CP, and CP ‘masqueraders’ (individuals with progressive symptoms). Twenty‐six per cent had a pathogenic/likely pathogenic variant in 1 of 13 genes, with the highest diagnostic yield in the CP masquerader category (3 out of 5, 60%) followed by the cryptogenic category (7 out of 24, 29%).^22^ This again indicates that patients with a CP presentation should be carefully phenotyped by an expert to identify ‘red flags’ for a possible genetic diagnosis.

Recently, Fehlings et al. presented results of WGS in 327 children with CP which included a comparison of children with (*n* = 37) and without (*n* = 290) pathogenic/likely pathogenic variants. Significant associations with pathogenic/likely pathogenic variants (*p* < 0.05) were found with the following phenotypic categories: normal brain MRI, cognitive impairment, communication difficulties, parental consanguinity, and term births. Crucially, the study also highlighted multiple known risk factors for CP in the group of children with pathogenic/likely pathogenic variants, suggesting that multifactorial pathways can lead to the clinical presentation of CP.^48^ Fahey et al. discussed this outcome in a previous review about the genetic contribution to CP.^21^ An earlier study using whole‐exome sequencing also found genetic causes in some children with clear environmental risk factors for CP.^49^ This raises interesting challenges for delineating patients with a likely genetic diagnosis for CP but an evolving phenotype due to the perinatal event, environmental exposure, or an interaction between these factors and genetic influences.

Undertaking WGS on all children with CP would pressurize already stretched laboratory services and would come at increased cost both in terms of sequencing and in clinician time (for consenting, obtaining trio samples, and interpretation). Increased genomic testing also increases the risk of incidental findings and issues around paternity which can lead to considerable distress for families and can be time‐consuming and complex for clinicians.

Ultimately, further research, discussion, and consultation are required. Some factors are associated with a high likelihood of a genetic diagnosis, for example consanguinity, or developmental regression. Some presentations associated with a genetic diagnosis, for example communication difficulties and cognitive impairment,^48^ are also seen in many children with or without CP. The presence of early environmental risk factors for CP is not as helpful as previously thought given the findings of Fehlings et al. discussed above.^48^ Finally, factors such as ‘term birth’ are of no discriminatory value in isolation. It seems prudent to continue to use good clinical assessment and judgement informed by the literature to guide genetic investigation until there are sufficient data to develop a robust guideline. It is also essential to combine decision‐making with prioritization based on assessment of clinical utility for the individual child and their family. We acknowledge that, in many countries of the world (often those with the highest rates of CP), resources do not allow for investigations such as WGS, and highlight the importance of retaining the ability to make a diagnosis of CP on clinical grounds alone.

Who should be the gatekeeper for genetic testing in CP where resources allow? In settings offering access to WGS, unless we reach the point that all children with CP receive genetic testing, it seems prudent that the decision to offer testing is in the hands of a specialist with knowledge of the clinical features associated with increased likelihood of a genetic cause. This question of clinical utility and gatekeeping of WGS is high on the list for many specialties, including learning disability psychiatry and community paediatrics where many patients are potentially eligible for WGS. Once a decision to offer testing is made, then ultimately the decision to proceed with testing lies with the patient or, for a child, those with parental responsibility.

## CONCLUSIONS

In this review we have briefly covered advances in our understanding of the aetiology of CP. We highlighted the pathophysiology using the *Wnt* pathway as an example, given the contribution of variants in the β‐catenin gene in this pathway to the condition CTNNB1 found in some children diagnosed with CP. We explored the relationship between genotype and phenotype in CP cohorts in the literature, listed ‘red flags’ for a genetic diagnosis, and demonstrated the need for a more robust genotype–phenotype reference data set. We discussed challenges to be addressed when considering whether to introduce genomic testing in CP. These include the wider implications of diagnostic labels; the need for accurate phenotyping; a clearer understanding of who should be tested; differentiation between monogenic causes and CP risk factors; logistics and cost. While it is premature to make recommendations for a national guideline, we provide perspectives on ways to integrate new understanding into care, pathways, suggestions for when to consider genetic testing, and the need for further research to inform consideration of CP as an indication for WGS testing.

## Supporting information


**Appendix S1:** Methods for literature review and term analysis.

## Data Availability

Data sharing not applicable ‐ no new data generated.
