# Supplementary material for: Genetic investigations in cerebral palsy
Source: Dev Med Child Neurol. 2024 Aug 29;67(2):177–85. doi: 10.1111/dmcn.16080 (PMC11695794; doi:10.1111/dmcn.16080)
Supplement: Supplementary file 1 — Appendix S1: Methods for literature review and term analysis. [file DMCN-67-177-s001.pdf]

Supplementary Information

Methods for literature review and term analysis:

We undertook a systematic review of the literature through a PubMed search limited to the past five years, extracting patient-level phenotype data for 199 patients (from 24 articles) diagnosed with monogenic diseases after being initially labelled as cerebral palsy (CP). The search string used is shown in Table S1. A five-year filter was used to ensure we collected articles reporting next generation sequencing technologies for genetic diagnosis. Rayyan software was used to filter articles based on title and abstract relevance [1]. The Preferred Reporting Items for Systematic Reviews and Meta-Analyses (PRISMA) approach was used during article screening for inclusion [2]. Exclusion criteria were articles not reporting patient-level data for patients with an initial diagnosis of CP who underwent genetic testing to identify a molecular genetic cause for their phenotype. A risk of bias assessment was performed using the Joanna Briggs Institute (JBI) Critical Appraisal Checklist for cohort studies [3]: we acknowledge the high heterogeneity of participants and cohort characteristics in studies reporting rare genetic diseases (articles listed in Table S2). We used the Human Phenotype Ontology (HPO) [4] to manually curate all phenotype data into a structured format for frequently occurring genes and compared this with available reference HPO annotations per gene (downloaded 18/02/2024) (**Figure 3A-B**). It is difficult to determine the frequency of HPO term association per gene through cohort data, therefore we analysed the frequency of each term per gene as shown in **Figure 3C** using only studies reporting patient-level data per gene. The overall term frequency in the dataset shown in the barplot (**Figure 3D**). We conducted these analyses and plots using R packages OntologyX [5], and ggplot2 [6].

Enriched terms were defined as those which are more prevalent in the published dataset than would be expected based on the general associations in the HPO database. These were investigated within the curated patient-level dataset by comparing against the downloaded HPO terms from hpo.jax.org for the same genes seen in the CP cohort datasets. The Fisher’s exact test was performed on relative proportions of these terms in

both datasets and the Benjamini-Hochberg method was used to control for multiple testing [7]. An adjusted *p*-value <0.05 was accepted as a significant result (Table S3).

Search number	Query	Filters	Search Details	Results
**5**	(diagnos*) AND (((cerebral palsy) AND ((genetic) OR (gene*))))	in the last 5 years	("diagnos*"[All Fields] AND (("cerebral palsy"[MeSH Terms] OR ("cerebral"[All Fields] AND "palsy"[All Fields]) OR "cerebral palsy"[All Fields]) AND ("genetic"[All Fields] OR "genetical"[All Fields] OR "genetically"[All Fields] OR "genetics"[MeSH Subheading] OR "genetics"[All Fields] OR "genetics"[MeSH Terms] OR "gene*"[All Fields]))) AND (y_5[Filter])	687
**4**	(diagnos*) AND (((cerebral palsy) AND ((genetic) OR (gene*))))		"diagnos*"[All Fields] AND (("cerebral palsy"[MeSH Terms] OR ("cerebral"[All Fields] AND "palsy"[All Fields]) OR "cerebral palsy"[All Fields]) AND ("genetic"[All Fields] OR "genetical"[All Fields] OR "genetically"[All Fields] OR "genetics"[MeSH Subheading] OR "genetics"[All Fields] OR "genetics"[MeSH Terms] OR "gene*"[All Fields]))	2,193
**3**	diagnos*		"diagnos*"[All Fields]	6,282,638
**1**	((cerebral palsy) AND ((genetic) OR (gene*)))		("cerebral palsy"[MeSH Terms] OR ("cerebral"[All Fields] AND "palsy"[All Fields]) OR "cerebral palsy"[All Fields]) AND ("genetic"[All Fields] OR "genetical"[All Fields] OR "genetically"[All Fields] OR "genetics"[MeSH Subheading] OR "genetics"[All Fields] OR "genetics"[MeSH Terms] OR "gene*"[All Fields])	5,790

Table S1: PubMed search strategy for genetic studies conducted in patients with cerebral palsy.

Title	Authors (year)	Cohort size
Genetic testing in individuals with cerebral palsy	May et al. (2021)[8]	150
Hidden etiology of cerebral palsy: genetic and clinical heterogeneity and efficient diagnosis by next-generation sequencing	Rosello et al. (2021)[9]	20
Genomics in Cerebral Palsy phenotype across the lifespan: Comparison of diagnostic yield between children and adult population	Al Zahrani et al. (2022)[10]	139
Genetic Testing Contributes to Diagnosis in Cerebral Palsy: Aicardi-Goutières Syndrome as an Example	Beysen et al. (2021)[11]	3
Yield of clinically reportable genetic variants in unselected cerebral palsy by whole genome sequencing	van Eyk et al. (2021)[12]	150
Dihydropteridine Reductase Deficiency - A Rare and Potentially Treatable Cause Mimicking Cerebral Palsy	Ribeiro et al. (2023)[13]	1
Mendelian etiologies identified with whole exome sequencing in cerebral palsy	Chopra et al. (2022)[14]	50
Diagnostic yield of chromosomal microarray and trio whole exome sequencing in cryptogenic cerebral palsy	Yechieli et al. (2022)[15]	45
Atypical cerebral palsy: genomics analysis enables precision medicine	Matthews et al. (2019)[16]	50
Hereditary spastic paraplegia initially diagnosed as cerebral palsy	Suchowersky et al. (2021)[17]	119
Arginase deficiency masked by cerebral palsy and coagulopathy-Three varied presentations of Latin American origin	Mills et al. (2023)[18]	3
Autosomal dominant ADAR c.3019G>A (p.(G1007R)) variant is an important mimic of hereditary spastic paraplegia and cerebral palsy	Jones et al. (2022)[19]	3
CTNNB1-related neurodevelopmental disorder mimics cerebral palsy: case report	Lee et al. (2023)[20]	2
ASXL3 De Novo Variant-Related Neurodevelopmental Disorder Presenting as Dystonic Cerebral Palsy	Švantnerová et al. (2022)[21]	1
MECP2 duplication syndrome initially misdiagnosed as cerebral palsy: a case report	Kim et al. (2023)[22]	1
Dystonic Cerebral Palsy Phenotype Due to GNAO1 Variant Responsive to Levodopa	Vasconcellos et al. (2023)[23]	1
COL4A1 mutation in an Indian child presenting as 'Cerebral Palsy' mimic	Shah et al. (2020)[24]	1
Niemann-Pick Disease Type C Misdiagnosed as Cerebral Palsy: A Case Report	Ko et al. (2019)[25]	1
A child with a novel DDX3X variant mimicking cerebral palsy: a case report	Hu et al. (2020)[26]	1
GNAO1 mutation presenting as dyskinetic cerebral palsy	Malaquias et al. (2019)[27]	1
PMM2-CDG T237M Mutation in a Patient with Cerebral Palsy-Like Phenotypes Reported from South India	Sreedevi et al. (2023)[28]	1
Pelizaeus-Merzbacher Disease with PLP1 Exon 1 Duplication, Previously Misdiagnosed as Cerebral Palsy: a Case Report	Lee et al. (2021)[29]	1
Infantile Onset of Spinocerebellar Ataxia Type 5 (SCA-5) in a 6 Month Old with Ataxic Cerebral Palsy	Rea et al. (2020)[30]	1
Mutations disrupting neuritogenesis genes confer risk for cerebral palsy	Jin et al. (2020)[31]	250

Table S2: Articles with patient-level phenotypic data, used to collect HPO terms. The cohort size refers to the overall cohort assessed in each study, however, not all these individuals had clinical data available for HPO curation. HPO data was manually curated for 199 individuals from the 24 articles.

HPO Term	Observed	Background	Adjusted *P* Value
Spastic diplegia	43	6	0.0000
Delayed gross motor development	36	10	0.0000
Spastic tetraplegia	37	17	0.0000
Abnormal brain morphology	21	2	0.0000
Athetoid cerebral palsy	15	0	0.0000
Progressive neurologic deterioration	18	3	0.0000
Intellectual disability	45	76	0.0000
Spastic hemiparesis	13	0	0.0000
Global developmental delay	43	77	0.0000
Dystonia	27	36	0.0000
Seizure	35	78	0.0000
Intellectual disability, moderate	17	17	0.0000
Left hemiplegia	7	0	0.0000
Neonatal asphyxia	7	0	0.0000
Very preterm birth	6	0	0.0001
Epileptic encephalopathy	11	10	0.0001
Hemiplegia	10	8	0.0002
Abnormal facial shape	13	17	0.0002
Motor regression	6	1	0.0003
Intraventricular hemorrhage	5	0	0.0004
Delayed speech and language development	20	45	0.0005
Neurodevelopmental delay	10	10	0.0005
Attention deficit hyperactivity disorder	12	16	0.0005
Periventricular leukomalacia	7	4	0.0010
Intellectual disability, profound	11	15	0.0011
Thin corpus callosum	7	5	0.0020
Deep cerebral white matter hyperintensities	4	0	0.0027
Spastic paraplegia	10	17	0.0077
Delayed fine motor development	4	1	0.0104
Encephalomalacia	4	1	0.0104
Hyperactive patellar reflex	4	1	0.0104
Periventricular white matter hyperintensities	5	3	0.0104
Stroke	5	3	0.0104
Elevated CSF neopterin level	3	0	0.0157
Extremely preterm birth	3	0	0.0157
Globus pallidus hypointensity on susceptibility-weighted imaging	3	0	0.0157
Hyperactive Achilles reflex	3	0	0.0157
Hypoxemia	3	0	0.0157
Impairment of activities of daily living	3	0	0.0157
Moderate to late preterm birth	3	0	0.0157
Progressive spastic quadriplegia	4	2	0.0218
Severe global developmental delay	8	16	0.0399

Generalized dystonia	4	3	0.0441
Loss of ambulation	5	6	0.0478

Table S3 Enriched HPO Terms which are more prevalent in the curated dataset. These resulted from comparing the observed rate (in curated dataset) to the background rate (in downloaded dataset). Adjusted p values shown following Fisher's exact test and BH adjustment.

References

1. Ouzzani M, Hammady H, Fedorowicz Z, Elmagarmid A. Rayyan—a web and mobile app for systematic reviews. *Systematic Reviews*. 2016/12/05 2016;5(1):210. doi:10.1186/s13643-016-0384-4
2. Liberati A, Altman DG, Tetzlaff J, et al. The PRISMA statement for reporting systematic reviews and meta-analyses of studies that evaluate healthcare interventions: explanation and elaboration. *BMJ*. 2009;339:b2700. doi:10.1136/bmj.b2700
3. Vardell E, Malloy M. Joanna Briggs Institute: An Evidence-Based Practice Database. *Medical Reference Services Quarterly*. 2013/10/01 2013;32(4):434-442. doi:10.1080/02763869.2013.837734
4. Gargano MA, Matentzoglu N, Coleman B, et al. The Human Phenotype Ontology in 2024: phenotypes around the world. *Nucleic Acids Res*. Jan 5 2024;52(D1):D1333-d1346. doi:10.1093/nar/gkad1005
5. Greene D, Richardson S, Turro E. ontologyX: a suite of R packages for working with ontological data. *Bioinformatics*. Apr 1 2017;33(7):1104-1106. doi:10.1093/bioinformatics/btw763
6. Wickham H. ggplot2: Elegant Graphics for Data Analysis. Springer-Verlag; 2022.
7. Benjamini Y, Hochberg Y. Controlling the false discovery rate: a practical and powerful approach to multiple testing. *Journal of the Royal statistical society: series B (Methodological)*. 1995;57(1):289-300.
8. May HJ, Fasheun JA, Bain JM, et al. Genetic testing in individuals with cerebral palsy. *Dev Med Child Neurol*. Dec 2021;63(12):1448-1455. doi:10.1111/dmcn.14948
9. Rosello M, Caro-Llopis A, Orellana C, et al. Hidden etiology of cerebral palsy: genetic and clinical heterogeneity and efficient diagnosis by next-generation sequencing. *Pediatr Res*. Aug 2021;90(2):284-288. doi:10.1038/s41390-020-01250-3
10. Al Zahrani H, Siriwardena K, Young D, Lehman A, Horvath GA, Goez H. Genomics in Cerebral Palsy phenotype across the lifespan: Comparison of diagnostic yield between children and adult population. *Mol Genet Metab*. Dec 2022;137(4):420-427. doi:10.1016/j.ymgme.2021.07.007
11. Beysen D, De Cordt C, Dielman C, et al. Genetic Testing Contributes to Diagnosis in Cerebral Palsy: Aicardi-Goutières Syndrome as an Example. *Front Neurol*. 2021;12:617813. doi:10.3389/fneur.2021.617813
12. van Eyk CL, Webber DL, Minoche AE, et al. Yield of clinically reportable genetic variants in unselected cerebral palsy by whole genome sequencing. *NPJ Genom Med*. Sep 16 2021;6(1):74. doi:10.1038/s41525-021-00238-0

13. Ribeiro M, Rebelo M, Pereira A, Antunes D, Ferreira AC, Jacinto S. Dihydropteridine Reductase Deficiency - A Rare and Potentially Treatable Cause Mimicking Cerebral Palsy. *Endocr Metab Immune Disord Drug Targets*. Nov 8 2023;doi:10.2174/0118715303279209231026044120
14. Chopra M, Gable DL, Love-Nichols J, et al. Mendelian etiologies identified with whole exome sequencing in cerebral palsy. *Ann Clin Transl Neurol*. Feb 2022;9(2):193-205. doi:10.1002/acn3.51506
15. Yechieli M, Gulsuner S, Ben-Pazi H, et al. Diagnostic yield of chromosomal microarray and trio whole exome sequencing in cryptogenic cerebral palsy. *J Med Genet*. Aug 2022;59(8):759-767. doi:10.1136/jmedgenet-2021-107884
16. Matthews AM, Blydt-Hansen I, Al-Jabri B, et al. Atypical cerebral palsy: genomics analysis enables precision medicine. *Genetics in Medicine*. 2019/07/01 2019;21(7):1621-1628. doi:10.1038/s41436-018-0376-y
17. Suchowersky O, Ashtiani S, Au PB, et al. Hereditary spastic paraplegia initially diagnosed as cerebral palsy. *Clin Park Relat Disord*. 2021;5:100114. doi:10.1016/j.prdoa.2021.100114
18. Mills SL, Roberts P, Ashfaq M, et al. Arginase deficiency masked by cerebral palsy and coagulopathy-Three varied presentations of Latin American origin. *JIMD Rep*. Nov 2023;64(6):434-439. doi:10.1002/jmd2.12397
19. Jones HF, Stoll M, Ho G, et al. Autosomal dominant ADAR c.3019G>A (p.(G1007R)) variant is an important mimic of hereditary spastic paraplegia and cerebral palsy. *Brain Dev*. Feb 2022;44(2):153-160. doi:10.1016/j.braindev.2021.10.001
20. Lee J, Yoo J, Lee S, Jang DH. CTNNB1-related neurodevelopmental disorder mimics cerebral palsy: case report. *Front Pediatr*. 2023;11:1201080. doi:10.3389/fped.2023.1201080
21. Švantnerová J, Minár M, Radová S, Kolníková M, Vlkovič P, Zech M. ASXL3 De Novo Variant-Related Neurodevelopmental Disorder Presenting as Dystonic Cerebral Palsy. *Neuropediatrics*. Oct 2022;53(5):361-365. doi:10.1055/s-0042-1750721
22. Kim TY, Lee SJ, Kim KM, Cho SR. MECP2 duplication syndrome initially misdiagnosed as cerebral palsy: a case report. *J Int Med Res*. Mar 2023;51(3):3000605231162452. doi:10.1177/03000605231162452
23. Vasconcellos LF, Soares VP, de Ricchezza LL. Dystonic Cerebral Palsy Phenotype Due to GNAO1 Variant Responsive to Levodopa. *Tremor Other Hyperkinet Mov (N Y)*. 2023;13:11. doi:10.5334/tohm.746
24. Shah SM, Patel DD. COL4A1 mutation in an Indian child presenting as 'Cerebral Palsy' mimic. *Indian J Radiol Imaging*. Oct-Dec 2020;30(4):500-503. doi:10.4103/ijri.IJRI_274_20
25. Ko EJ, Sung IY, Yoo HW. Niemann-Pick Disease Type C Misdiagnosed as Cerebral Palsy: A Case Report. *Ann Rehabil Med*. Oct 2019;43(5):621-624. doi:10.5535/arm.2019.43.5.621
26. Hu L, Xin X, Lin S, et al. A child with a novel DDX3X variant mimicking cerebral palsy: a case report. *Ital J Pediatr*. Jun 29 2020;46(1):88. doi:10.1186/s13052-020-00850-3
27. Malaquias MJ, Fineza I, Loureiro L, Cardoso L, Alonso I, Magalhães M. GNAO1 mutation presenting as dyskinetic cerebral palsy. *Neurol Sci*. Oct 2019;40(10):2213-2216. doi:10.1007/s10072-019-03964-7
28. Sreedevi N, Swapna N, Maruthy S, Meghavathi HS, Sylvester C. PMM2 -CDG T237M Mutation in a Patient with Cerebral Palsy-Like Phenotypes Reported from South India. *Glob Med Genet*. Jun 2023;10(2):105-108. doi:10.1055/s-0043-1769494

29. Lee SJ, Kim TY, Hong S, Byun J, Cho SR. Pelizaeus-Merzbacher Disease with PLP1 Exon 1 Duplication, Previously Misdiagnosed as Cerebral Palsy: a Case Report. *Brain Neurorehabil*. Jul 2021;14(2):e20. doi:10.12786/bn.2021.14.e20
30. Rea G, Tirupathi S, Williams J, Clouston P, Morrison PJ. Infantile Onset of Spinocerebellar Ataxia Type 5 (SCA-5) in a 6 Month Old with Ataxic Cerebral Palsy. *Cerebellum*. Feb 2020;19(1):161-163. doi:10.1007/s12311-019-01085-7
31. Jin SC, Lewis SA, Bakhtiari S, et al. Mutations disrupting neuritogenesis genes confer risk for cerebral palsy. *Nat Genet*. Oct 2020;52(10):1046-1056. doi:10.1038/s41588-020-0695-1
